# Metabolic Reprogramming in Response to Alterations of Mitochondrial DNA and Mitochondrial Dysfunction in Gastric Adenocarcinoma

**DOI:** 10.3390/ijms23031857

**Published:** 2022-02-06

**Authors:** Tzu-Ching Chang, Hui-Ting Lee, Siao-Cian Pan, Shih-Han Cho, Chieh Cheng, Liang-Hung Ou, Chia-I Lin, Chen-Sung Lin, Yau-Huei Wei

**Affiliations:** 1 Institute of Cellular and System Medicine, National Health Research Institutes, Miaoli County 350, Taiwan; ctching9@gmail.com or; 2 School of Life Science, National Taiwan Normal University, Taipei 116, Taiwan; safjacoco@yahoo.com.tw (S.-H.C.); 40505015e@gapps.ntnu.edu.tw (C.C.); surg90139@gmail.com (L.-H.O.); 3 Division of Allergy, Immunology and Rheumatology, Department of Internal Medicine, Mackay Memorial Hospital, Taipei 104, Taiwan; htlee1228@gmail.com; 4 Department of Medicine, Mackay Medical College, New Taipei City 252, Taiwan; 5 Center for Mitochondrial Medicine and Free Radical Research, Changhua Christian Hospital, Changhua City 500, Taiwan; k.elantris@gmail.com; 6 School of Medicine, College of Medicine, National Yang Ming Chiao Tung University, Taipei 112, Taiwan; 7 Division of General Surgery, Department of Surgery, Taipei Hospital, Ministry of Health and Welfare, New Taipei City 242, Taiwan; 8 Division of Pathology, Taipei Hospital, Ministry of Health and Welfare, New Taipei City 242, Taiwan; chiai0914@gmail.com; 9 Division of Thoracic Surgery, Department of Surgery, Taipei Hospital, Ministry of Health and Welfare, New Taipei City 242, Taiwan; 10 Center for General Education, Kainan University, Taoyuan City 338, Taiwan

**Keywords:** copy number, D310 mutation, gastric adenocarcinoma (GAC), metabolic reprogramming, mitochondrial DNA (mtDNA), mitochondrial transcription factor A (TFAM), prognosis

## Abstract

We used gastric cancer cell line AGS and clinical samples to investigate the roles of mitochondrial DNA (mtDNA) alterations and mitochondrial respiratory dysfunction in gastric adenocarcinoma (GAC). A total of 131 clinical samples, including 17 normal gastric mucosa (N-GM) from overweight patients who had received sleeve gastrectomy and 57 paired non-cancerous gastric mucosae (NC-GM) and GAC from GAC patients who had undergone partial/subtotal/total gastrectomy, were recruited to examine the copy number and D310 sequences of mtDNA. The gastric cancer cell line AGS was used with knockdown (KD) mitochondrial transcription factor A (TFAM) to achieve mitochondrial dysfunction through a decrease of mtDNA copy number. Parental (PT), null-target (NT), and TFAM-KD-(A/B/C) represented the parental, control, and TFAM knocked-down AGS cells, respectively. These cells were used to compare the parameters reflecting mitochondrial biogenesis, glycolysis, and cell migration activity. The median mtDNA copy numbers of 17 N-GM, 57 NC-GM, and 57 GAC were 0.058, 0.055, and 0.045, respectively. The trend of decrease was significant (*p* = 0.030). In addition, GAC had a lower mean mtDNA copy number of 0.055 as compared with the paired NC-GM of 0.078 (*p* < 0.001). The mean mtDNA copy number ratio (mtDNA copy number of GAC/mtDNA copy number of paired NC-GM) was 0.891. A total of 35 (61.4%) GAC samples had an mtDNA copy number ratio ≤0.804 (*p* = 0.017) and 27 (47.4%) harbored a D310 mutation (*p* = 0.047), and these patients had shorter survival time and poorer prognosis. After effective knockdown of TFAM, TFAM-KD-B/C cells expressed higher levels of hexokinase II (HK-II) and v-akt murine thymoma viral oncogene homolog 1 gene (*AKT*)-encoded AKT, but lower levels of phosphorylated pyruvate dehydrogenase (p-PDH) than did the NT/PT AGS cells. Except for a higher level of p-PDH, the expression levels of these proteins remained unchanged in TFAM-KD-A, which had a mild knockdown of TFAM. Compared to those of NT, TFAM-KD-C had not only a lower mtDNA copy number (*p* = 0.050), but also lower oxygen consumption rates (OCR), including basal respiration (OCR_BR_), ATP-coupled respiration (OCR_ATP_), reserve capacity (OCR_RC_), and proton leak (OCR_PL_)(all with *p* = 0.050). In contrast, TFAM-KD-C expressed a higher extracellular acidification rate (ECAR)/OCR_BR_ ratio (*p* = 0.050) and a faster wound healing migration at 6, 12, and 18 h, respectively (all with *p* = 0.050). Beyond a threshold, the decrease in mtDNA copy number, the mtDNA D310 mutation, and mitochondrial dysfunction were involved in the carcinogenesis and progression of GACs. Activation of PDH might be considered as compensation for the mitochondrial dysfunction in response to glucose metabolic reprogramming or to adjust mitochondrial plasticity in GAC.

## 1. Introduction

Gastric adenocarcinoma (GAC) is an aggressive malignancy that originates from the gastric mucosa (GM) and is one of the leading causes of cancer-related deaths in Taiwan [1]. Most oncologists have paid attention to the mutations or oncological effects of nuclear DNA (nDNA) [2], and a few have investigated the roles of mitochondrial DNA (mtDNA) and mitochondrial biogenesis and their roles in the carcinogenesis, progression, and prognosis of GAC [3,4,5].

In addition to serving as the powerhouse that generates ATP in human cells [6,7], mitochondria are regarded as a multi-functional hub that produces precursors for the biosynthesis of amino acids, lipids, and nucleotides, and controls cell death [8]. These features confer the mitochondria with the capacity of metabolic reprogramming or mitochondrial plasticity to regulate the fate of human cells [9]. From the classical viewpoint of energy production by using glucose as the major fuel, 1 mole of glucose could generate approximately 36–38 moles of ATP after complete metabolism, including glycolysis in the cytoplasm (an oxygen-independent process), and the tricarboxylic acid (TCA) cycle as well as oxidative phosphorylation (OXPHOS) in the mitochondria [6,7]. In glycolysis, glucose can produce pyruvate by glycolytic enzymes with hexokinase (HK) as the first step [10]. Converted by pyruvate dehydrogenase (PDH), pyruvate, which is considered a central metabolic node [11], joins the TCA cycle in the form of acetyl-CoA to release high energy intermediates, the reduced flavin adenine dinucleotide (FADH_2_), and reduced nicotinamide adenine dinucleotide (NADH). Under the premise of sufficient oxygen supply, FADH_2_ and NADH would generate ATP through electron transport and OXPHOS [6,7]. If tissues suffer from hypoxia or mitochondrial damages, pyruvate would be reduced to lactate by lactate dehydrogenase (LDH) in the cytoplasm rather than entering the mitochondria. This process is so-called anaerobic glycolysis and only two moles of ATP per mole of glucose are generated [6,7]. 

Mitochondria are unique organelles because they have their own genome, the mtDNA, which is distinct from the nDNA. As a rule, a human cell possesses approximately a few hundred to thousands of copies of mtDNA, and they are replicated independently from the cell cycle within the scattered mitochondria. Undertaken by fission and fusion, these replicated/or existing mtDNA copies could segregate or gather as nucleoids in the inner membrane of daughter mitochondria to form the dynamic mitochondrial network [6,12]. Human mtDNA (Available online: http://www.mitomap.org, accessed on 4 November 2021), with a size of 16569 base-pairs (bp), is a circular and double-stranded DNA containing an inner light (L) strand and an outer heavy (H) strand, which harbor 37 compact genes coding for 13 polypeptides, 2 rRNAs, and 22 tRNAs. The 13 polypeptides constitute a small but indispensable part of the OXPHOS machinery, and the 2 rRNAs plus 22 tRNAs are required for protein synthesis inside the mitochondria. In the non-coding region, the displacement loop (D-loop) contains essential elements required for the transcription and replication of mtDNA, including promoters of both H and L strands and the replication origin of the H strand [6,12]. Except for the 13 polypeptides, all the other mitochondrial proteins, OXPHOS machinery included, are encoded by genes in nDNA because of lateral (horizontal) transfer during the evolution of mtDNA [13]. The OXPHOS system has a dual gene origin, and the nDNA-encoded subunits are translated in the cytosol prior to import into the mitochondria and are then assembled with the mtDNA-encoded subunits inside mitochondria, which implies that mtDNA plays a determining role in mitochondrial function. Theoretically, the more mtDNA copies a tissue cell has, the higher the respiratory function and more ATP production would be ensured [6,12].

Different from the heterozygotic nature of nDNA, the human mtDNA is transmitted through the maternal lineage. As a result, the genomic sequences of all mtDNA copies are considered identical in the post-mitotic infant tissues [14], a condition called homoplasmy. In the aged human tissues or affected tissues of patients with certain mitochondrial diseases, mtDNA variants would co-exist with the wild-type mtDNA molecules, and the homoplasmy is disrupted and results in heteroplasmy [14]. Because of a lack of protection by histones, replication by the low fidelity of DNA polymerase gamma and its location at the site near electron transport with a high concentration of reactive oxygen species (ROS), mtDNA accumulates damages much faster than does the nDNA [6,14,15,16]. Throughout the entire mtDNA genome, the D-loop is a hot spot for mutation, especially at the nucleotide position (np) 310 [17]. There is a polycytosine (poly-C) tract (PCT) consisting of repeated mono-C between np 303 and np 316 with thymidine (T) interposed at np 310 (-C_303_CCCCCC_309_-T_310_-C_311_CCCCC_316_-). Generally, the repeated C number after T remains constant as 6. Often the C number before T is 7 (C_7_) or 8 (C_8_), but 3, 4, 5, 6, 9, 10, and 11 (C_3_, C_4_, C_5_, C_6_, C_9_, C_10_, C_11_, varied degrees of C deletion or insertion) have also been reported. These variants are referred to as D310 polymorphism or PCT length polymorphism (Available online: http://www.mitomap.org, accessed on 4 November 2021). Compared to the D310 variants of the paired non-cancer counterparts, any sequence alterations (insertion, deletion, or proportion shift) that occur in the cancer tissues are defined as D310 mutations [18,19]. 

Defects in mtDNA integrity are usually manifested as the mtDNA harboring damage/alteration/mutation or depletion of mtDNA. These mtDNA defects limit the capacity of mitochondria to meet the energy demand of affected tissues. To maintain the integrity of mtDNA, several proteins including RNA polymerase, mitochondrial transcription factor A (TFAM), mitochondrial single-stranded-DNA-binding protein A/B, DNA polymerase gamma, nuclear respiratory factors (NRF) 1/2, and peroxisome proliferators-activated receptor-gamma coactivator-1 (PGC-1α) are involved in the homeostasis of the mtDNA pool and mitochondrial biogenesis [6,12]. Among them, TFAM plays a pivotal role because it regulates both the replication and transcription of mtDNA through its binding to the D-loop of mtDNA.

Dr. Otto Warburg was the first who described glucose metabolic changes in human cancers in the 1950′s [20]. He reported that human cancers exhibited the phenomenon of avid glucose uptake, amplified glycolysis, and profound lactate production even in the presence of an ample supply of oxygen, a situation called aerobic glycolysis. He also found that the proportion of ATP generated by glycolysis is increased but that generated by oxidative metabolism is decreased. This metabolic shift of aerobic glycolysis in human cancers is termed the Warburg effect [20,21]. Dr. Warburg proposed that respiratory function is impaired in human cancers and might be involved in carcinogenesis [20,21].

To examine the mitochondrial alterations in GAC, we addressed the following issues in this study. First, we analyzed the alterations of mtDNA, including the copy number and D310 variants, in human GAC to dissect their biological and clinical implications. Next, we knocked down the TFAM expression to decrease the mtDNA copy number and to achieve mitochondrial respiratory dysfunction in a GAC cell line and then evaluated the consequence on cancer glucose metabolism and the aggressiveness of cancer cells. We intended to have a comprehensive understanding of the role of mtDNA alterations and mitochondrial dysfunction in human GAC.

## 2. Results

### 2.1. Distributions of D310 Variants and mtDNA Copy Numbers of Analyzed Samples

Overall, we analyzed 131 samples including 17 normal gastric mucosa (N-GM), 57 non-cancerous gastric mucosa (NC-GM,) and 57 gastric adenocarcinomas (GAC) in this study. Concerning the D310 characteristics of the 131 samples, 45 (34.4%) had homoplasmy and 64 (48.9%) harbored C_8_TC_6_ as the major variant; there was a mean of 1.9 variants. There were no significant differences in the D310 homoplasmy (*p* = 0.324/0.547), the major D310 variant (*p* = 0.578/0.105), and a number of D310 variants (*p* = 0.829/0.179) between the 57 paired NC-GM and GAC samples and among the 17 N-GM, 57 NC-GM, and 57 GAC samples (Table 1, upper part).

The detailed information about the D310 variants of the 57 paired NC-GM and GAC are listed in Supplementary Table S1. Compared to the D310 variants of paired NC-GM, there were four types of D310 alterations classified in corresponding GAC (Supplementary Table S1 and Figure S1), including (1) Type I, Homoplasmic to homoplasmic alteration, without D310 mutation, *n* = 17, (2) Type II, Heteroplasmic to heteroplasmic alteration, without D310 mutation, *n* = 13, (3) Type III, Heteroplasmic to heteroplasmic alteration, with D310 mutation, *n* = 22, and (4) Type IV, Heteroplasmic to homoplasmic alteration, with D310 mutation, *n* = 5. Finally, 27 (47.4%) GAC patients were found to harbor a D310 mutation in their GAC tissues (Table 1, upper part).

The mean and median mtDNA copy numbers of the 131 samples (17 N-GM, 57 NC-GM, and 57 GAC) were 0.066 (0.060, 0.078, and 0.055) and 0.050 (0.058, 0.055, and 0.045), respectively. Compared to the paired NC-GM, the 57 GAC had a lower mean mtDNA copy number (0.055 ± 0.045 vs. 0.078 ± 0.076, *p* < 0.001). Among the 17 N-GM, 57 NC-GM, and 57 GAC, the median mtDNA copy numbers decreased from 0.058 to 0.055 and then 0.045 (*p* = 0.030). Using 0.050, the median copy number of the 131 samples, as a cutoff value, we found that the proportions of N-GM, NC-GM, and GAC harboring mtDNA copy number exceeding 0.050 (>0.050) decreased progressively from 64.7% (11/17) to 57.9% (33/57) and then to 36.8% (21/57) (*p* = 0.013) (Table 1, upper part).

The mtDNA copy number ratios of the 57 GAC were 0.891 ± 0.968. A total of 29 GAC patients were alive at the time of follow-up, and they had a higher mtDNA copy number ratio than did the other 28 who were deceased (1.081 ± 1.314 vs. 0.694 ± 0.273, *p* = 0.032). We then tested various cutoff points for the mtDNA copy number ratio on receiver operating characteristic (ROC) curves (area under the curve, AUC = 0.666, 95%CI = 0.523–0.808, *p* = 0.032) to distinguish their survival status among 57 GAC patients and found that the mtDNA copy number ratio of 0.804 had the highest Youden index of 0.338 (sensitivity = 0.786, specificity = 0.552, Supplementary Figure S2). We defined those with a mtDNA copy number ratio ≤ 0.804 as the low mtDNA copy number ratio group (*n* = 35, 61.4%) and those with a mtDNA copy number ratio > 0.804 as the high mtDNA copy number ratio group (*n* = 22, 38.6%), respectively (Table 1, lower part).

### 2.2. Demographic Data of the 57 GAC Patients

A total of 57 GAC patients (37 men, 64.9%) with a mean age of 67.1 years were analyzed (Table 2). One (1.8%) underwent a partial gastrectomy, 44 (77.2%) had a subtotal gastrectomy, and 12 (21.1%) received a total gastrectomy. Their mean maximal tumor diameter was 4.4 cm, and there were 12 (21.1%), 6 (10.5%), 27 (47.4%), and 12 (21.1%) in T1, T2, T3, and T4 status, 19 (33.3%), 3 (5.3%), 13 (22.8%), and 22 (38.6%) in N0, N1, N2, and N3 status, and 44 (77.2%) and 13 (22.8%) in M0 and M1 status, respectively. Eight GAC (14.0%) harbored well-differentiated tumor cells, 23 (40.4%) had moderately differentiated tumor cells, and 26 (45.6%) owned poorly differentiated tumor cells. Concerning the conditions of cancer invasion, 4 (7.0%) had resection margin invasion, 35 (61.4%) showed lymphovascular invasion, and 35 (61.4%) had perineural invasion, respectively. Regarding the mtDNA alterations, 35 (61.4%) had a low mtDNA copy number ratio and 27 (47.4%) had a D310 mutation. Their mean survival time and follow-up periods were 44.2 and 22.5 months, respectively (Table 2).

### 2.3. Prognostic Variables and Their Hazard Ratios (HRs)

**Figure 1:**
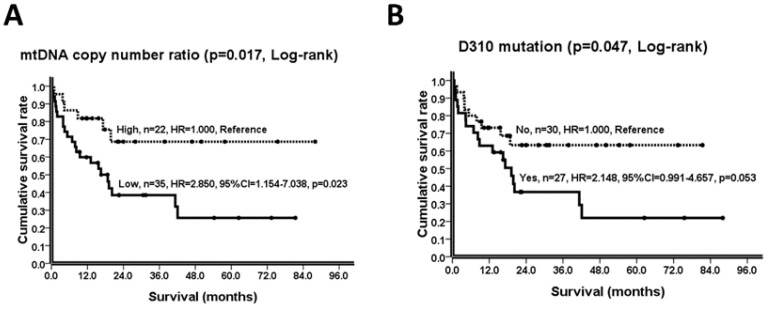
Kaplan-Meier survival curves, *p*-values (Log-rank test), and their HRs (including 95% CI, Cox proportional-hazards regression, univariate) about the prognostic roles of (**A**) mtDNA copy number ratio, high vs. low and (**B**) D310 mutation, no vs. yes in GAC patients are illustrated. GAC patients harboring a low mtDNA copy number ratio (*p* = 0.017) or D310 mutation (*p* = 0.047) had poorer prognosis. CI: confidence interval; GAC: gastric adenocarcinoma; HR: hazard ratio; High: mtDNA copy number ratio, >0.804; Low: mtDNA copy number ratio, ≤0.804; No: without the D310 mutation; Yes: with the D310 mutation.

As shown in Table 3 (left part), T-status (T1 vs. T2 vs. T3 vs. T4, *p* = 0.008), N-status (N0 vs. N1 vs. N2 vs. N3, *p* = 0.049), M-status (M0 vs. M1, *p* < 0.001), cancer cell differentiation (well vs. moderate/poor, *p* = 0.091), maximal tumor diameter (≤4 vs. >4 cm, *p* = 0.002), lymphovascular invasion (no vs. yes, *p* = 0.015), perineural invasion (no vs. yes, *p* = 0.002), mtDNA copy number ratio (high vs. low, *p* = 0.017; Figure 1A), and D310 mutation (no vs. yes, *p* = 0.047; Figure 1B) are possible prognostic variables to distinguish survival.

In the univariate Cox proportional hazards regression model, patients with advanced T status (T4, HR = 10.679, *p* = 0.027; T3, HR = 13.468, *p* = 0.012; T2, HR = 4.490, *p* = 0.220; T1, HR = 1.000, Reference), advanced N status (N3, HR = 3.925, *p* = 0.009; N2, HR = 2.813, *p* = 0.078; N1, HR = 1.804, *p* = 0.591; N0, HR = 1.000, Reference), M1 status (M1, HR = 4.648, *p* < 0.001; M0, HR = 1.000, Reference), moderate/poor cancer cell differentiation (moderate/poor, HR = 3.236, *p* = 0.111; well, HR = 1.000, Reference), maximal tumor diameter > 4 cm (> 4cm, HR = 3.273, *p* = 0.004; ≤ 4 cm, HR = 1.000, Reference), lymphovascular invasion (yes, HR = 2.922, *p* = 0.020; no, HR = 1.000, Reference), perineural invasion (yes, HR = 4.184, *p* = 0.005; no, HR = 1.000, Reference), mtDNA copy number ratio (low, HR = 2.850, *p* = 0.023; high, HR = 1.000, Reference; Figure 1A), and the D310 mutation (yes, HR = 2.148, *p* = 0.053; no, HR = 1.000, Reference; Figure 1B) tended to have higher HRs (Table 3, middle part).

Under the Cox proportional hazards regression model with multivariate analysis, patients with M1 status (M1, HR = 2.902, 95%CI = 0.991–8.496, *p* = 0.052; M0, HR = 1.000, Reference) was identified as an independent poor prognostic variable with elevated HR (Table 3, right part).

### 2.4. Cancer Pathological Characteristics and mtDNA Alterations

Advanced T-status (*p* = 0.064), advanced N-status (*p* = 0.070), M1-status (*p* = 0.009), and moderate/poor cancer cell differentiation (*p* = 0.002) were found to be highly associated with those who had a low mtDNA copy number ratio (Table 4, left). Additionally, advanced T-status (*p* = 0.007), maximal tumor diameter >4 cm (*p* = 0.006), and perineural invasion (*p* = 0.062) were highly related to those who had a D310 mutation (Table 4, right).

Based on the above results, we concluded that (1) from N-GM to NC-GM and then GAC, mtDNA copy numbers decreased in a stepwise manner (Table 1), (2) GAC patients harboring mtDNA alterations, either a low mtDNA copy number ratio or a D310 mutation, had poorer prognosis (Table 3, Figure 1), and (3) these mtDNA alterations were highly associated with either advanced T-/ or N-/ or M-status or maximal tumor diameter >4 cm (Table 4). These findings have led us to speculate that mtDNA alterations may be involved in the carcinogenesis, progression, and prognosis of GACs.

Although the mtDNA copy number of GAC is lower than that of the NC-GM counterpart with an mtDNA copy ratio of 0.891, the optimal cutoff to distinguish survival is 0.804. Like other human diseases, it is possible that there is a mtDNA copy number threshold that could be tolerated by GAC patients [22]. Beyond the critical point, a more malignant or compensation process of the GAC seemed to be involved. 

### 2.5. Effect of TFAM Knockdown in AGS Cells and Their Alterations

**Figure 2:**
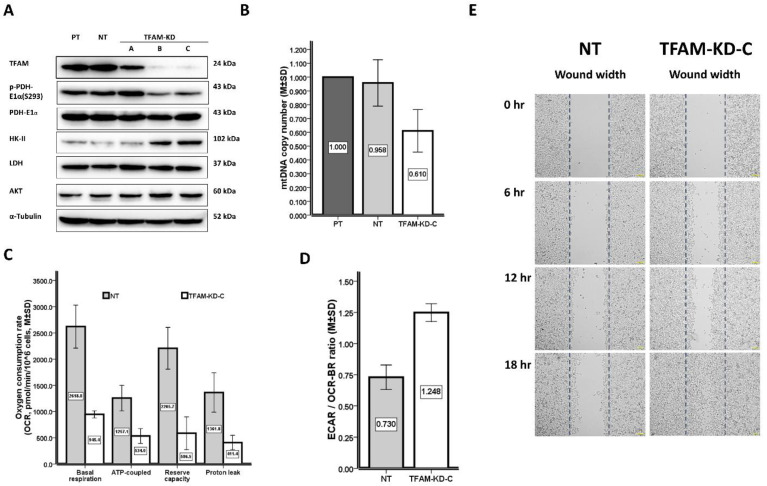
(**A**) In the left two lanes, the Western blot shows that clones NT and PT had similar TFAM/PDH-E1α/p-PDH-E1α(S293)/HK-II/LDH/AKT expression levels. The α-tubulin is used as the internal control. In the first row, clones TFAM-KD-B/C expressed obviously lower levels and clone TFAM-KD-A expressed a mildly lower level of TFAM than did clones NT/PT. In the second row, clones TFAM-KD-B/C expressed lower levels of p-PDH-E1α(S293), but clone TFAM-KD-A expressed a higher level of p-PDH-E1α(S293) than did clones NT/PT. In the third row, clones TFAM-KD-A/B/C expressed similar levels of PDH-E1α to those of clones NT/PT. In the fourth row, clones TFAM-KD-B/C expressed higher levels of HK-II, and clone TFAM-KD-A expressed a similar level of HK-II to those of clones NT/PT. In the fifth row, clones TFAM-KD-A/B/C expressed similar levels of LDH to those of clones NT/PT. In the sixth row, clones TFAM-KD-B/C expressed higher levels of AKT, and clone TFAM-KD-A expressed a similar level of AKT to those of clones NT/PT. (**B**) The clone TFAM-KD-C had a lower mtDNA copy number (0.610 ± 0.154 vs. 0.958 ± 0.168, *p* = 0.050) than did the clone NT. (**C**) The clone TFAM-KD-C had lower OCRs (pmol/min/10^6^ cells), including those for basal respiration (OCR_BR_)(945.4 ± 67.5 vs. 2618.8 ± 410.9, *p* = 0.050), ATP-coupled (OCR_ATP_)(534.0 ± 139.3 vs. 1257.1 ± 243.8, *p* = 0.050), reserve capacity (OCR_RC_)(586.5 ± 309.8 vs. 2205.7 ± 397.9, *p* = 0.050), and proton leak (OCR_PL_)(411.4 ± 134.5 vs. 1361.8 ± 374.8, *p* = 0.050), than did the clone NT. (**D**) The clone TFAM-KD-C had a higher ECAR/OCR_BR_ ratio (1.248 ± 0.071 vs. 0.730 ± 0.098, *p* = 0.050) than did the clone NT. (**E**) The clone TFAM-KD-C had a shorter wound width (μm) at 6 hr (333.8 ± 19.7 vs. 459.0 ± 13.6, *p* = 0.050), 12 hr (251.9 ± 2.0 vs. 361.7 ± 3.1, *p* = 0.050), and 18 hr (115.7 ± 11.1 vs. 330.0 ± 22.0, *p* = 0.050), respectively, than did the clone NT. NT: Null target; PT: Parental; TAFM: mitochondrial transcription factor A; PDH-E1α: pyruvate dehydrogenase E1α subunit; p-PDH-E1α (293): phosphorylated pyruvate dehydrogenase E1α subunit; HK-II: hexokinase II; LDH: lactate dehydrogenase; AKT: v-akt murine thymoma viral oncogene homolog 1 gene (*AKT*)-encoded AKT; mtDNA: mitochondrial DNA; OCR: oxygen consumption rate; ECAR: extracellular acidification rate; hr: hour.

TFAM knockdown (KD) was performed in clones TFAM-KD-A/B/C. Western blot (WB) demonstrated that the knockdown efficiency was obvious in TFAM-KD-B/C, but mild in TFAM-KD-A as compared to that of NT (Figure 2A & Supplementary Figure S3A).

Concerning the dynamic changes between PDH-E1α and p-PDH-E1α(S293), we found that TFAM-KD-A/B/C expressed similar PDH-E1α levels to that of NT regardless of the degrees of TFAM knockdown (Figure 2A and Supplementary Figure S3C). It is important to mention that TFAM-KD-A expressed a higher and TFAM-KD-B/C a lower p-PDH-E1α(S293) level than did the NT after a mild and an obvious TFAM knockdown, respectively (Figure 2A and Supplementary Figure S3B). In addition, we demonstrated that TFAM-KD-A expressed a higher and TFAM-KD-B/C a lower p-PDH-E1α(S293)/ PDH-E1α ratio than did the NT after a mild and an obvious TFAM knockdown, respectively (Figure 2A and Supplementary Figure S3D) These findings suggest that an activated PDH, reflected by less phosphorylation, might have occurred to enhance pyruvate turnover and to compensate for the suppressed mitochondrial function after an obvious TFAM knockdown.

Concerning the alterations of glycolytic enzymes and their regulators, we found (1) TFAM-KD-A expressed similar levels of HK-II, LDH, and AKT to that of NT after a mild TFAM knockdown, and (2) TFAM-KD-B/C expressed higher levels of HK-II and AKT, but the level of LDH was similar to that of NT after an obvious TFAM knockdown (Figure 2A and Supplementary Figure S3E–G). These observations imply that glycolysis was up regulated after an obvious TFAM knockdown.

Of note, the TFAM/PDH-E1α/p-PDH-E1α(S293)/HK-II/LDH/AKT expression levels were similar between NT and PT (Figure 2A and Supplementary Figure S3A–C,E–G), suggesting that the above findings of clones TFAM-KD-A/B/C were caused by the selective consequences of TFAM knockdown rather than off-target effects.

The TFAM-KD-C had a lower mtDNA copy number (0.610 ± 0.154 vs. 0.958 ± 0.168, *p* = 0.050) and lower OCRs (pmol/min/10^6^ cells), including OCR_BR_ (945.4 ± 67.5 vs. 2618.8 ± 410.9, *p* = 0.050), OCR_ATP_ (534.0 ± 139.3 vs. 1257.1 ± 243.8, *p* = 0.050), OCR_RC_ (586.5 ± 309.8 vs. 2205.7 ± 397.9, *p* = 0.050), and OCR_PL_ (411.4 ± 134.5 vs. 1361.8 ± 374.8, *p* = 0.050), but higher ECAR/OCR_BR_ ratios (1.248 ± 0.071 vs. 0.730 ± 0.098, *p* = 0.050) than did the NT (Table 5 and Figure 2B–D). We have thus demonstrated a glucose metabolic shift in AGS cells due to mitochondrial dysfunction after an effective TFAM knockdown.

TFAM-KD-C had a shorter wound width (μm), at 6 h (333.8 ± 19.7 vs. 459.0 ± 13.6, *p* = 0.050), 12 h (251.9 ± 2.0 vs. 361.7 ± 3.1, *p* = 0.050), and 18 h (115.7 ± 11.1 vs. 330.0 ± 22.0, *p* = 0.050), respectively, than did the NT (Table 5 and Figure 2E). The proliferation rates of TFAM-KD-C and NT cells were similar during growth on day 1~3 (<72 h), but TFAM-KD-C cells had a lower proliferation rate than did NT cells on day 4~5 (>96 h) (Supplementary Figure S4). This suggests that the higher migration ability of TFAM-KD-C cells is a result of TFAM knock-down rather than the difference in proliferation rate.

In the cell line study, we observed a decrease in the mtDNA copy number induced by an effective TFAM knockdown that resulted in (1) lower OCRs, but a higher ECAR/OCR_BR_ ratio, (2) higher AKT, higher HK-II, but lower p-PDH-E1α(293) protein expression levels and (3) higher horizontal migration activity (Table 5, Figure 2 and Figure S3). These findings led us to propose that mitochondrial dysfunction caused by a decrease of mtDNA copy number can induce metabolic reprogramming of glucose utilization, including enhanced glycolysis, less inhibition of (phosphorylated) PDH, and a more aggressive phenotype in the AGS gastric cancer cell line [23].

## 3. Discussion

Since the observations made by Dr. Warburg, the likelihood of mitochondrial dysfunction in human cancers has been appraised since the 1950′s [24]. Generally speaking, the respiratory function of mitochondria is highly related to the integrity of mtDNA. A decrease of the mtDNA copy number or a mutation in mtDNA implies a decline of mitochondrial respiration [6,25,26].

A decrease of the mtDNA copy number has been reported in several human cancers, including renal cancer [27], hepatic cancer [28], breast cancer [29], and gastric cancer [4,30], which has been confirmed in this study. Importantly, we found a progressive decrease of the mtDNA copy number from the gastric mucosa of normal controls to the pathological normal gastric mucosa and then to the gastric adenocarcinoma of patients with gastric cancer (Table 1). This observation suggests that a decrease of the mtDNA copy number may be involved in the carcinogenesis of gastric cancer [31]. Additionally, a decrease in the mtDNA copy number has been reported in the progression of esophageal squamous cell carcinomas [32] and non-small lung cancers [33,34], poor prognosis of breast cancer [35], the development of Borrmann’s types III/IV GAC [4], and advanced stage III/IV GAC [30], as well as advanced T-/N-/M-status and poorer prognosis of GAC, observed in the current study (Table 3 and Table 4; Figure 1A). Taken together, a decrease in the mtDNA copy number plays a role in the carcinogenesis, progression, and poor prognosis of GAC.

We reported that 27 (47.4%) of 57 GAC patients carried D310 mutations (Table 1), and they had a poorer prognosis (Table 3; Figure 1B). Moreover, the frequency of the D310 mutation was increased with T-status or related to tumor diameter >4 cm during tumor progression (Table 4). Likewise, D-loop/or D310 mutations were detected in lung cancer [36], esophageal squamous cell carcinoma [18], and breast cancer [19,29], and have been implicated in the progression and/or poor prognosis of breast cancer [19,29], lung cancer [37], and colorectal cancer [38]. These observations indicate that D310 mutations may play a role in the development, progression, and poor prognosis of GAC [3].

The role of mtDNA integrity in the pathogenesis of human diseases has been evaluated for many years [39]. Because the replication of mtDNA is independent of and unsynchronized to the cell cycle, the DNA replication machinery in mitochondria seems not to be the only regulator controlling mtDNA integrity inside the rapidly proliferating cancer cells. The dynamics of mitochondrial membrane and morphology, i.e., the switch between fission and fusion, has been emphasized because of their association with mtDNA integrity and distinct glucose metabolic patterns [40]. An adequate mitochondrial fusion is required for the maintenance of mtDNA integrity and the execution of OXPHOS, otherwise, the occurrence of deletion/mutation or depletion of mtDNA, or impaired OXPHOS would follow [26]. On the contrary, mitochondrial fission is characteristic of loose mitochondrial cristae and it is advantageous for cells to upregulate aerobic glycolysis with reduced OXPHOS [41]. During carcinogenesis, transformed cancer cells tend to exhibit an increase of fission but a decrease of fusion [26]. Our data showed a decrease in the mtDNA copy number and a mutant mtDNA during carcinogenesis and progression of GAC. The role of mitochondrial morphology in GAC deserves further elucidation in the future.

To evaluate the consequences of decreased mtDNA copy number in human cancers, we have focused on the TFAM-mtDNA pathway in the past 10 or more years. We knocked down TFAM to decrease the mtDNA copy number in several human cancer cell lines, including esophageal squamous cell carcinoma (ESCC) [42], renal cell carcinoma (RCC) [27], colorectal cancer (CRC) [43], and non-small cell lung cancer (NSCLC) [33] as well as GAC (Figure 2 & Supplementary Figure S3; Table 5) in the current study. A decrease in the mtDNA copy number did cause a decrease in mitochondrial biogenesis as reflected by lower expression levels of mtDNA-encoded polypeptides in ESCC, RCC, CRC, and NSCLC cells or lower rates of oxygen consumption in ESCC, RCC, CRC, and GAC (Figure 2C) cells. In contrast, amplified glycolysis was verified through an increase of the expression of glycolytic enzymes or proteins in ESCC, RCC, CRC, and GAC (Figure 2A) cells, an increase of lactate production in ESCC and CRC cells, and an increase of ECAR in RCC or elevated ECAR/OCR_BR_ ratio in RCC and GAC (Figure 2D) cells. Despite a lower mtDNA copy number ratio being identified as a poor prognostic variable for GAC patients in the current study, M1 status remained an independent variable (Figure 1A; Table 3) and the M1 status was highly related to a lower mtDNA copy number ratio (Table 4). We speculated the amplified glycolysis induced by mitochondrial dysfunction may participate in cancer metastases. Epithelial-to-mesenchymal transition (EMT) is a key step in cancer metastases [44]. It has been demonstrated that either mitochondrial dysfunction, evidenced by mtDNA depletion [45], or amplified glycolysis, evidenced by an increase of HK-II expression [46], is involved in the initiation of EMT [47]. Likewise, we found that the GAC cancer cell line had a higher migration activity while expressing higher HK-II and lower mtDNA copy numbers after an obvious TFAM knockdown (Figure 2). Although glycolysis is an inefficient way to generate ATP, it offers an advantage to cancer cells via an offshoot of the pentose phosphate pathway for nucleotide biosynthesis, or an acidic environment to help cancers escape from immune surveillance [48,49] or the initiation of EMT [47].

The above model of TFAM knockdown seems to be a robust platform to validate the Warburg effect concerning the amplification of glycolysis and mitochondrial dysfunction in human cancers. However, some confounding results happened. During TFAM knockdown, we found an increase in migration activity in RCC, NSCLC, and GAC (Figure 2E) cells [27,33], but a decrease in migration activity in ESCC and CRC cells [22,38]. Although we did not evaluate the protein levels of TFAM in GAC patients’ specimens in this study, relevant results have been reported in other human cancers. In a previous study, we found that a low level of TFAM protein expression was associated with the progression and poor prognosis in NSCLC [33]. In contrast, Mo et al. [50] reported that a high level of TFAM protein expression was associated with the progression of bladder cancer. The Human Protein Atlas stated that the low expression of TFAM is related to unfavorable outcomes in renal cell carcinoma and ovarian cancer, but a favorable outcome in endometrial cancer (please refer to the website, Available online: https://www.proteinatlas.org/; accessed on 4 November 2021). There must be some unresolved puzzles about the Warburg effect in human cancers if we only think about the capacity of energy production. Cancer cells require not only the supply of ATP, but also the abundant biosynthesis of macromolecules during carcinogenesis and tumor progression. Of note, cancer mitochondria could take over the above task because they are capable of generating ATP and the production of precursors for the biosynthesis of amino acids, lipids, and nucleotides, or controlling cell death [8]. Regarding the classical Warburg effect, mitochondrial metabolic reprogramming was regarded as a new hallmark of cancers [51], because it would offer mitochondrial plasticity to cope with the different environments of different cancers.

To achieve the hallmark of cancer cells, the importance of the pyruvate/PDH/acetyl-CoA pathway deserves emphasis [52,53]. Through the oxidation by active un-phosphorylated PDH, pyruvate would be converted to acetyl-CoA in the mitochondria. After entering the TCA cycle, acetyl-CoA enables mitochondria either to generate ATP to meet the energy demand or to derive fatty acids for the biosynthesis of phospholipids or amino acids for the biosynthesis of proteins, which are cornerstones for tumor growth, progression, and invasion [11,54]. Interestingly, as compared to the control, our results revealed that PDH became more phosphorylated after a mild TFAM knockdown but less phosphorylated after an obvious TFAM knockdown (Figure 2A). We speculated that activated PDH generates acetyl-CoA to support the biosynthesis of macromolecules or membrane phospholipids in gastric cancer (Figure 2A). The newly synthesized membrane phospholipids are required for mitochondrial fusion/fission, which adjusts the cancer cells to adapt to the decrease of mtDNA copy number after TFAM knockdown and influence cancer cell migration [55,56].

Of note, the pyruvate/PDH/acetyl-CoA pathway is a one-way and irreversible reaction. Nevertheless, the activity of PDH is reversible and is tightly regulated by the balance between phosphorylation, executed by pyruvate dehydrogenase kinase (PDK), and dephosphorylation, executed by pyruvate dehydrogenase phosphatase (PDP) [57]. In other words, relative PDK/PDP activities might influence cellular metabolic flexibility [58] and offer mitochondrial plasticity in human cancers [59]. Taken together, evaluation of the expression levels of p-PDH, PDH, PDK, and PDP is warranted in future studies to underscore the metabolic reprogramming and mitochondrial plasticity in human GAC or other cancers.

The oncogenic and glycolysis-enhancing effects of AKT have been widely discussed in the field of cancer research [60]. Interestingly, we found an increase in the protein expression level of AKT and a higher migration activity after TFAM knockdown in GAC cells (Figure 2A,E). The above phenomenon has also been reported in RCC cells [27] and head and neck cancers [61]. It is possible that PI3K/AKT plays an important role in the regulation of PDH to trigger glucose metabolic reprogramming and more invasive activities in human cancers [60]. In addition, mitochondrial dysfunction might induce specific integrin-β1 N-glycosylation patterns, thereby promoting the fibronectin-binding of cancer cells and enhancing cell migration [62]. However, the molecular mechanism warrants further study in the future.

## 4. Materials and Methods

### 4.1. Study Participants, Tissue Preparation, and DNA Extraction

A total of 57 GAC patients who had received primary partial/subtotal/total gastrectomy with D2 lymph node dissection [1] and 17 overweight patients who had received a sleeve gastrectomy between January 2012 and December 2019 in Taipei Hospital, Ministry of Health and Welfare (MOHW), New Taipei City, Taiwan were retrospectively recruited. Their pathological T-/N-/M-status and cancer stages were designated according to the AJCC 8th edition [63]. Reviewed by an experienced pathologist (C-I Lin), representative areas on pathological slides harboring the GAC and the paired non-cancerous gastric mucosa (NC-GM) of GAC patients and normal gastric mucosa (N-GM) of overweight patients were selected. Thin sections about 5 μm in thickness were cut from the corresponding tissue blocks for DNA extraction. After the de-wax and re-hydration procedures, the tissue sections were mixed with 500 μL of QuickExtract^TM^ DNA extraction solution (Epicenter, Madison, WI, USA) to extract total cellular DNA at 65 °C for 3 h as described [18,19,33]. The DNA samples thus obtained were kept at −20 °C until use.

### 4.2. Human GAC Cell Line, TFAM Knockdown, Stable Clone Selection, and Cellular DNA/Protein Extractions

The AGS (ATCC^®^ CRL-1739^™^) human GAC cell line [64], cultured in RPMI 1640 medium (Gibco, Grand Island, NY, USA) containing 10% fetal bovine serum (FBS, Gibco, Grand Island, NY, USA) and 1% antibiotics (Penicillin G and streptomycin sulfate, Biological Industries, Kibbutz Beit HaEmek, Israel), was used to conduct the cell experiments described in this study.

A small hairpin RNA (sh-RNA) was obtained from the National RNAi Core Facility of Academia Sinica, Taiwan (Available online: http://rnai.genmed.sinica.edu.tw) and was used to knock down TFAM expression as described [27,33]. A plasmid derived from the pLKO.1 backbone harboring a Puromycin-resistant gene and sh-RNA with a specific sequence of 5′-CGTTTATGTAGCTGAAAGATT-3′ against TFAM mRNA was packaged into lentiviral particles to infect AGS cells. For comparison, a null target (NT) sequence 5′-TCAGTTAACCACTTTTT-3′ was used as the control [27,33]. After two multiplicity of infection (MOI) of viral infection for 48 h, the culture medium was changed to that containing Puromycin (1.4 μg/mL, lethal dosage for AGS cells). Stable clones were established after 14 days of Puromycin selection and they were confirmed to have no signs of apoptosis. AGS cells (parental cell, named PT) harboring the pLKO.1-sh-TFAM plasmids were named TFAM-KD. AGS cells harboring the pLKO.1-sh-NT plasmids were named NT.

Total cellular DNA was extracted by phenol/chloroform as described and kept at −20 °C until use [27,33]. Total cellular proteins were harvested by using a lysis buffer (50 mM Tris-HCl, 0.25% sodium deoxycholate, 150 mM NaCl, 1 mM EDTA, 1% Triton X-100, and 1% NP-40, pH 7.4) containing 1% of protease inhibitors (Roche Applied Sciences, Penzberg, Germany) as described and kept at −80 °C until use [27,33].

### 4.3. Quantification of mtDNA and nDNA Copies and Analysis of mtDNA Copy Number

Quantitative real-time polymerase chain reaction (Q-PCR) using Fast SYBR™ Green Master Mix (Thermo Fisher Scientific, Baltics UAB, Vilnius, Lithuania) was used to detect the threshold cycles (Ct values) at different concentrations of genomic DNA, which were applied to establish the equations to calculate the mtDNA copies and nDNA copies. Total cellular DNA of AGS cells was serially diluted 4-fold from 100 to 0.024414 ng/μL for Q-PCR to detect Ct values of mtDNA and nDNA, respectively. The sequences of primers used for mtDNA (tRNA^Leu(UUR)^ gene) and nDNA (18S rRNA gene) amplification were mtF3212: 5’-CACCCAAGAACAGGGTTTGT-3’/mtR3319: 5’-TGGCCATGGGA TTGTTGTTAA-3’; and 18SF1546: 5′-TAGAGGGACAAGTGGCGTTC-3′/18SR1650: 5′-CGCTGAGCCAGTCAGTGT-3′, respectively [18,19,27,33]. The equations established for the calculation of mtDNA copies and nDNA copies were: the Ct value of sample mtDNA = (−3.3166) × log (mtDNA copies of sample DNA / mtDNA copies of AGS cells) + 21.976 (*R*^2^ = 0.9991), and the Ct value of sample nDNA = (−3.1220) × log (nDNA copies of sample DNA/nDNA copies of AGS cells) + 27.407 (*R*^2^ = 0.9975), respectively (Supplementary Figure S5).

The mtDNA copy number was defined as the total mtDNA copies / total nDNA copies as described [18,19,27,33]. Q-PCR was performed to calculate mtDNA and nDNA copies. For each reaction, 1 μL of sample DNA (10 ng/μL) was amplified in a 10-μL mixture containing 0.5 μL of each primer (20 μM, mtF, and mtR for mtDNA; 18SF and 18SR for nDNA), 5 μL of Fast SYBR™ Green Master Mix (Thermo Fisher Scientific, Baltics UAB, Vilnius, Lithuania), and 3 μL of PCR grade water. Simultaneously, 1 μL of DNA from AGS cells (1 ng/μL) and PCR grade water were included as the positive and negative controls, respectively. The PCR conditions were set as follows: hot start at 95 °C for 10 min followed by 45 cycles of 95 °C for 15 s and 60 °C for 60 s. The mtDNA copies and nDNA copies of the sample DNA relative to those of AGS cells were calculated based on the above equations. The mtDNA copy numbers of clinical samples or cell clones were measured after adjusting the mtDNA copy number of the AGS cell as 1.000. Each experiment was done in duplicate to get the average mtDNA copy number for clinical samples and was repeated for three independent batches in the cell line study (N = 3).

To determine the changes of mtDNA copy numbers between the GAC and paired NC-GM samples, we defined the mtDNA copy number ratio as the mtDNA copy number of GAC divided by the mtDNA copy number of paired NC-GM of each GAC patient.

### 4.4. Sequencing of the D310 Region of mtDNA

The D310 region of mtDNA was PCR-amplified and then subjected to direct sequencing [18,19]. Each 50 μL PCR reaction contained 10 μL of PCR Hot-Start Master Mix II (5X, Scientific Biotech Corp., Taipei, Taiwan), 33 μL of PCR-grade H_2_O, 1 μL of each primer (H76-1: 5′-CACGCGATAGCATTGCGA-3′ and L335: 5′-TAAGTGCTGTGGCCAGA AGC-3′), and 5 μL of sample DNA (10 ng/μL). The PCR procedures included hot start at 95 °C for 5 min, 40 cycles of 95 °C for 15 s, 58 °C for 15 s and 72 °C for 30 s, and a final extension at 72 °C for 7 min. Confirmed by electrophoresis on a 3% agarose gel, the PCR products were subjected to direct sequencing (MB Mission Biotech, Taipei, Taiwan). The D310 sequences, including patterns of homoplasmy or heteroplasmy, the number of D310 variants, and the predominant D310 variant were determined as described [18,19]. Compared to the D310 sequence of the paired NC-GM, any shift in GAC was defined as a D310 mutation [18,19]. Examples are illustrated in Supplementary Figure S1.

### 4.5. Determination of Relative Protein Expression Levels

The relative protein expression levels in a cell lysate were determined by Western blot (WB). An aliquot of 30 μg of total cellular proteins was separated on a 10% SDS-PAGE and then blotted onto a piece of BioTrace™ polyvinylidene difluoride (PVDF) membrane (Pall Corp., Pensacola, FL, USA). Non-specific bindings were blocked with 5% skimmed milk in a Tris-buffered saline Tween-20 (TBST) buffer (50 mM Tris-HCl, 150 mM NaCl, and 0.1% Tween-20, pH 7.4). The membrane was then subjected to specific primary antibodies (protein name, company, dilution fold, molecular weight) against target proteins, including mitochondrial transcription factor A (TFAM, Cell Signaling, 1:1000, 24 kD), pyruvate dehydrogenase E1 alpha (PDH-E1α, Santa Cruz, 1:1000, 43 kD), phosphorylated PDH-E1α(S293)(p-PDH-E1α(S293), Abcam, 1:1000, 43 kD), hexokinase II (HK-II, Proteintech, 1:1000, 102 kD), lactate dehydrogenase (LDH, Abcam, 1:2000, 37 kD), and v-akt murine thymoma viral oncogene homolog 1 gene (*AKT*)-encoded AKT (AKT, Cell Signaling, 1:1000, 60 kD). Alpha-tubulin (α-tubulin, Proteintech, 1:5000, 52 kD) was used as the internal control. The membrane was then incubated with horseradish peroxidase (HRP)-conjugated secondary antibody and the protein bands were visualized on an X-ray film (Fujifilm) by using ECL reagents (GE Healthcare, Chalfont, UK). Protein band intensities were quantified by using the Image J software (National Institutes of Health, Bethesda, MD, USA, Available online: https://imagej.nih.gov/ij/, accessed on 26 November 2021) and the data of NT cells were adjusted to 1.00. Each experiment was repeated using three batches of the cultured cells (N = 3).

### 4.6. Analysis of Bioenergetic Parameters by the XF^e^-24 Analyzer

An aliquot of 40,000 NT or TFAM-KD-C cells were harvested to detect the oxygen consumption rate (OCR), including basal respiration OCR (OCR_BR_), ATP-coupled OCR (OCR_ATP_), reserve capacity OCR (OCR_RC_), and proton leak OCR (OCR_PL_), after the administration of oligomycin (Complex V inhibitor, 1 µM), carbonyl cyanide 4-(trifluoromethoxy)-phenylhydrazone (0.3 µM FCCP, an uncoupling agent of mitochondrial respiration to achieve the maximal respiration rate) and rotenone/antimycin A (Complex I/III inhibitor, 0.5 µM) in order on a Seahorse XF^e^-24 Analyzer (Seahorse Bioscience, Billerica, MA, USA) as described [25,27,64]. Before the addition of oligomycin, the extracellular acidification rate (ECAR) of the cells was determined according to the instructions of the manufacturer. To determine the trends of metabolic shift, we calculated the ECAR/OCR_BR_ ratio for comparison [25,27]. Experiments were performed using three independent batches of the cell culture (N = 3). All the data are presented as mean ± SD after adjustment to pmol/min/10^6^ cells for OCR and mpH/min/10^6^ cells for ECAR.

### 4.7. Wound Healing Migration Assay

Cell migration was analyzed by a wound healing assay using ibidi culture inserts (ibidi GmbH, Munich, Germany) as described [65]. The insert contains two reservoirs separated by a 500-µm thick wall. The culture insert was placed into a 24-well culture plate and about 6 × 10^4^ cells were added into each reservoir for overnight culture. A 500-µm gap was created after removing the culture insert. The images of the cells were photographed at different time points (0, 6, 12, and 18 h) by using an Olympus IX83 microscope. Quantification was carried out by using tools of the CellSens Imaging Software to measure the width of the wound. Experiments were performed using three independent batches of the cell culture (N = 3). All the data are presented as mean ± SD.

### 4.8. Statistical Analysis

SPSS statistical software version 17 (SPSS Inc., Chicago, IL, USA) was used for data analysis. The survivals of GAC patients were calculated from the date of surgery to the date of death or last follow-up in December 2019, and the survival curves were plotted by the Kaplan–Meier method. The Log-rank test was used to compare the survival probabilities among different levels of possible prognostic variables, and the univariate Cox proportional hazards regression method was used to investigate their relative hazard ratios (HRs). Variables associated with the survival probability at a significance level of 0.1 or less (≤0.1) in the Log-rank test were included in the multivariate Cox proportional hazards regression model. The continuous variables between or among groups were compared using paired *t*-test/Wilcoxon signed-rank test, *t*-test/Mann-Whitney *U* test, or ANOVA/Kruskal-Wallis H/Jonckheere-Terpstra test when appropriate. Categorical variables between or among groups were compared by using the Chi-square (*χ*^2^) test, Fisher exact test, or Chi-square (*χ*^2^) test for trend (linear-by-linear association) when appropriate.

To subgroup the 57 GAC patients, the optimal cutoff point of the mtDNA copy number ratio for survival prognosis was plotted by the receiver operating characteristic (ROC) curves to figure out the highest Youden index (Youden index = sensitivity + specificity − 0-pds#gfvv1) [66]. A significant difference is considered when the p-value is less than 0.05.

## 5. Conclusions

In conclusion, we demonstrated in this study that mtDNA alterations and mitochondrial dysfunction in GAC may play a role in the Warburg effect and enhanced aggressiveness conferred by metabolic reprogramming of glucose utilization.

## Figures and Tables

**Table 1:** Distributions of the mtDNA copy numbers, D310 variants, and their alterations among 17 N-GM and 57 paired NC-GM and GAC.

DNA Variables		Subjects/Analyzed Tissue Samples				
	Overall (*n* = 131, 100.0%)	Controls (*n* = 17)	GAC Patients (*n* = 57)		*p*-Value	
		N-GM (*n* = 17, 100.0%)	NC-GM (*n* = 57, 100.0%)	GAC (*n* = 57, 100.0%)	a	b
D310 pattern						
Homoplasmy	45 (34.4%)	6 (35.3%)	17 (29.8%)	22 (38.6%)	0.324 *	0.547 ****
Heteroplasmy	86 (65.6%)	11 (64.7%)	40 (70.2%)	35 (61.4%)		
Major D310 variant						
C_7_TC_6_	47 (35.9%)	4 (23.5%)	20 (35.1%)	23 (40.4%)	0.578 *	0.105 ****
C_8_TC6	64 (48.9%)	8 (47.1%)	29 (50.9%)	27 (47.4%)		
Others	20 (15.3%)	5 (29.4%)	8 (14.0%)	7 (12.3%)		
Number of D310 variants	1.9 ± 1.0	2.4 ± 1.9	1.9 ± 0.7	1.9 ± 0.8	0.829 **	0.179 ***
D310 mutation						
No				30 (52.6%)		
Yes				27 (47.4%)		
mtDNA copy number						
Mean ± SD	0.066 ± 0.060	0.060 ± 0.030	0.078 ± 0.076	0.055 ± 0.045	<0.001 **	0.071 ***
Median	0.050	0.058	0.055	0.045		0.030 ***
≤0.050	66 (50.4%)	6 (35.3%)	24 (42.1%)	36 (63.2%)		0.013 ****
>0.050	65 (49.6%)	11(64.7%)	33 (57.9%)	21 (36.8%)		
mtDNA copy number ratio				Mean ± SD	*p*-value *****	
Overall (*n* = 57, 100.0%)				0.891 ± 0.968		
Survival status						
Alive (*n* = 29, 50.9%)				1.081 ± 1.314	0.032	
Dead (*n* = 28, 49.1%)				0.694 ± 0.273		
Subgroups						
Low (≤0.804, *n* = 35, 61.4%)				0.579 ± 0.138	<0.001	
High (>0.804, *n* = 22, 38.6%)				1.387 ± 1.431		

a, analyzed between the paired NC-GM and GAC of the 57 GAC patients; b, analyzed among the 17 N-GM, 57 NC-GM, and 57 GAC; * χ^2^ test; ** Wilcoxon signed-rank test or paired *t*-test when appropriate; *** Kruskal-Wallis *H* test, Jonckheere-Terpstra test or Analysis of Variance (ANOVA) when appropriate; **** Linear by linear association (χ^2^ test for trend); ***** *t*-test or Mann-Whitney *U* test when appropriate; GAC: Gastric adenocarcinoma; GM: Gastric mucosa; N-GM: Normal gastric mucosa; NC-GM: Non-cancerous gastric mucosa; mtDNA copy number ratio = mtDNA copy number of GAC/mtDNA copy number of paired NC-GM of each GAC patient.

**Table 2:** Demographic data of the 57 GAC patients.

Variables	Case Number (%)/Mean ± SD
Sex	
Male/Female	37 (64.9)/20 (35.1)
Age (years)	67.1 ± 13.4
Type of resection	
Partial/subtotal/total gastrectomy	1 (1.8)/4 (77.2)/12 (21.1)
Pathological finding	
T-status *	
T1/T2/T3/T4	12 (21.1)/6 (10.5)/27 (47.4)/12 (21.1)
N-status *	
N0/N1/N2/N3	19 (33.3)/3 (5.3)/13 (22.8)/22 (38.6)
M-status *	
M0/M1	44 (77.2)/13 (22.8)
Differentiation	
Well/moderate/poor	8 (14.0)/23 (40.4)/26 (45.6)
Maximal tumor diameter (cm)	4.4 ± 2.5
≤4 cm/>4 cm	30 (52.6)/27 (47.4)
Resection margin invasion	
No/Yes	53 (93.0)/4 (7.0)
Lymphovascular invasion	
No/Yes	22 (38.6)/35 (61.4)
Perineural invasion	
No/Yes	22 (38.6)/35 (61.4)
mtDNA copy number ratio	0.891 ± 0.968
High (>0.804)/Low (≤0.804)	22 (38.6%)/35 (61.4%)
D310 mutation	
No/Yes	30 (52.6%)/27 (47.4%)
Survivals (months)	44.2 ± 5.7
Follow-up periods (months)	22.5 ± 21.9

* American Joint Committee on Cancer (AJCC), 8th edition.

**Table 3:** Prognostic variables and their hazard ratios of the 57 GAC patients.

	Survival Differences	Log-Rank	Univariate Cox’s Regression		Multivariate Cox’s Regression	
Variables/Case Number	Survival (95% CI), Months	*p*-Value	HRs (95% CI)	*p*-Value	HRs (95% CI)	*p*-Value
Sex		0.584				
Male (*n* = 37)	42.2 (29.1−55.3)		1.000			
Female (*n* = 20)	36.0 (24.4−47.7)		0.794 (0.347−1.819)	0.585		
Age		0.410				
≤65 (*n* = 27)	46.8 (31.5−62.1)		1.000			
>65 (*n* = 30)	40.0 (25.6−54.5)		1.375 (0.642−2.946)	0.412		
Type of resection		0.400				
Partial gastrectomy (*n* = 1)	7.9 (7.9−7.9)		3.649 (0.473−28.126)	0.214		
Subtotal gastrectomy (*n* = 44)	44.1 (32.3−55.8)		1.000			
Total gastrectomy (*n* = 12)	37.9 (15.9−59.9)		1.209 (0.511−2.862)	0.666		
Pathological finding						
T-status *		0.008				
T1 (*n* = 12)	66.7 (54.4−79.0)		1.000		1.000	
T2 (*n* = 6)	43.3 (21.4−65.1)		4.490 (0.407−49.541)	0.220	2.112 (0.183−27.412)	0.567
T3 (*n* = 27)	23.9 (13.1−34.7)		13.468 (1.789−101.421)	0.012	2.554 (0.180−36.301)	0.489
T4 (*n* = 12)	36.7 (13.5−59.9)		10.679 (1.310−87.034)	0.027	1.572 (0.092−26.766)	0.755
N-status *		0.049				
N0 (*n* = 19)	54.7 (40.7−68.6)		1.000		1.000	
N1 (*n* = 3)	58.7 (11.8−105.6)		1.804 (0.210−15.480)	0.591	1.306 (0.080−21.287)	0.852
N2 (*n* = 13)	38.1 (17.5−58.8)		2.813 (0.890-8.887)	0.078	1.370 (0.178-10.548)	0.762
N3 (*n* = 22)	19.9 (12.5−27.2)		3.925 (1.407−10.944)	0.009	0.784 (0.103−5.975)	0.815
M-status *		<0.001				
M0 (*n* = 44)	53.0 (40.4−65.6)		1.000		1.000	
M1 (*n* = 13)	8.8 (4.7−12.9)		4.648 (2.074−10.418)	<0.001	2.902 (0.991−8.496)	0.052
Cancer cell differentiation		0.091				
Well (*n* = 8)	57.0 (36.1−77.9)		1.000		1.000	
Moderate/Poor (*n* = 49)	40.2 (28.1−52.3)		3.236 (0.765−13.693)	0.111	2.300 (0.356−14.850)	0.382
Maximal tumor diameter		0.002				
≤4 cm (*n* = 30)	56.6 (43.3−70.0)		1.000		1.000	
>4 cm (*n* = 27)	26.9 (13.3−40.6)		3.273 (1.474−7.270)	0.004	2.185 (0.721−6.625)	0.167
Resection margin invasion		0.345				
No (*n* = 53)	46.4 (34.8−58.0)		1.000			
Yes (*n* = 4)	23.5 (1.1−45.8)		1.771 (0.532−5.895)	0.352		
Lymphovascular invasion		0.015				
No (*n* = 22)	62.5 (45.3−79.7)		1.000		1.000	
Yes (*n* = 35)	30.9 (19.1−42.7)		2.922 (1.180−7.236)	0.020	0.671 (0.104−4.313)	0.674
Perineural invasion		0.002				
No (*n* = 22)	68.4 (53.4−83.4)		1.000		1.000	
Yes (*n* = 35)	25.0 (14.3−35.7)		4.184 (1.558−11.239)	0.005	2.708 (0.690−10.626)	0.153
mtDNA copy number ratio		0.017				
High (*n* = 22)	63.7 (47.2−80.1)		1.000		1.000	
Low (*n* = 35)	31.6 (19.8−43.4)		2.850 (1.154−7.038)	0.023	1.597 (0.530−4.812)	0.406
D310 mutation		0.047				
No (*n* = 30)	54.5 (40.9−68.1)		1.000		1.000	
Yes (*n* = 27)	31.6 (17.9−45.3)		2.148 (0.991−4.657)	0.053	1.825 (0.724−4.601)	0.202

* American Joint Committee on Cancer (AJCC), 8th edition; mtDNA copy number ratio ≤ 0.804 defined as “Low” mtDNA copy number ratio; mtDNA copy number ratio > 0.804 defined as “High” mtDNA copy number ratio; CI: confidence interval; GAC: gastric adenocarcinoma; HR: hazard ratio.

**Table 4:** Cancer pathological characteristics and mtDNA alterations.

	mtDNA Copy Number Ratio			D310 Mutation		
Variables (Case Number, %)	Low (*n* = 35)	High (*n* = 22)	*p*-Value *	Yes (*n* = 27)	No (*n* = 30)	*p*-Value *
T-status			0.064			0.007
T1 (*n* = 12, 100.0%)	4 (33.3%)	8 (66.7%)		2 (16.7%)	10 (83.3%)	
T2 (*n* = 6, 100.0%)	4 (66.7%)	2 (33.3%)		2 (33.3%)	4 (66.7%)	
T3 (*n* = 27, 100.0%)	19 (70.4%)	8 (29.6%)		15 (55.6%)	12 (44.4%)	
T4 (*n* = 12,100.0%)	8 (66.7%)	4 (33.3%)		8 (66.7%)	4 (33.3%)	
N-status			0.070			0.153
N0 (*n* = 19, 100.0%)	9 (47.4%)	10 (52.6%)		7 (36.8%)	12 (63.2%)	
N1 (*n* = 3, 100.0%)	2 (66.7%)	1 (33.3%)		1 (33.3%)	2 (66.7%)	
N2 (*n* = 13, 100.0%)	7 (53.8%)	6 (46.2%)		6 (46.2%)	7 (53.8%)	
N3 (*n* = 22, 100.0%)	17 (77.3%)	5 (22.7%)		13 (59.1%)	9 (40.9%)	
M-status			0.009			0.464
M0 (*n* = 44, 100.0%)	23 (52.3%)	21 (47.7%)		22 (50.0%)	22 (50.0%)	
M1 (*n* = 13, 100.0%)	12 (92.3%)	1 (7.7%)		5 (38.5%)	8 (61.5%)	
Differentiation			0.002			0.547
Well (*n* = 8, 100.0%)	1 (12.5%)	7 (87.5%)		3 (37.5%)	5 (52.5%)	
Moderate/Poor (*n* = 49, 100.0%)	34 (69.4%)	15 (30.6%)		24 (49.0%)	25 (51.0%)	
Maximal tumor diameter			0.752			0.006
≤4 cm (*n* = 30, 100.0%)	19 (63.3%)	11 (36.7%)		9 (30.0%)	21 (70.0%)	
>4 cm (*n* = 27, 100.0%)	16 (59.3%)	11 (40.7%)		18 (66.7%)	9 (33.3%)	
Lymphovascular invasion			0.161			0.439
No (*n* = 22, 100.0%)	11 (50.0%)	11 (50.0%)		9 (40.9%)	13 (59.1%)	
Yes (*n* = 35, 100.0%)	24 (68.6%)	11 (31.4%)		18 (51.4%)	17 (48.6%)	
Perineural invasion			0.161			0.062
No (*n* = 22, 100.0%)	11 (50.0%)	11 (50.0%)		7 (31.8%)	15 (68.2%)	
Yes (*n* = 35, 100.0%)	24 (68.6%)	11 (31.4%)		20 (57.1%)	15 (42.9%)	

* *χ*^2^ test or linear by linear association (*χ*^2^ test for trend); mtDNA copy number ratio ≤ 0.804 defined as “Low” mtDNA copy number ratio; mtDNA copy number ratio > 0.804 defined as “High” mtDNA copy number ratio.

**Table 5:** The differences in mitochondrial DNA copy number, wound width, oxygen consumption rate, and extracellular acidification rate in the AGS PT, NT, and TFAM-KD-C clones.

	AGS Cell Lines				
Variables (Mean ± SD)	PT (*n* = 3)	NT (*n* = 3)	TFAM-KD-C (*n* = 3)	*p* ^a^	*p* ^b^
mtDNA copy number	1.000 ± 0.000	0.958 ± 0.168	0.610 ± 0.154	0.700	0.050
Oxygen consumption rate (OCR, pmol/min/10^6^ cells)					
Basal respiration (OCR_BR_)		2618.8 ± 410.9	945.4 ± 67.5		0.050
ATP-coupled (OCR_ATP_)		1257.1 ± 243.8	534.0 ± 139.3		0.050
Reserve capacity (OCR_RC_)		2205.7 ± 397.9	586.5 ± 309.8		0.050
Proton leak (OCR_PL_)		1361.8 ± 374.8	411.4 ± 134.5		0.050
Extracellular acidification rate (ECAR, mpH/min/10^6^ cells)		1884.7 ± 80.2	1183.3 ± 153.5		0.050
ECAR/OCR_BR_ ratio		0.730 ± 0.098	1.248 ± 0.071		0.050
Wound width (μm)					
0 h		493.1 ± 8.8	491.4 ± 14.3		1.000
6 h		459.0 ± 13.6	333.8 ± 19.7		0.050
12 h		361.7 ± 3.1	251.9 ± 2.0		0.050
18 h		330.0 ± 22.0	115.7 ± 11.1		0.050

*p*^a^ compared between PT and NT cells, Mann-Whitney *U* test; *p* ^b^ compared between NT and TFAM-KD-C cells, Mann-Whitney *U* test.

## Data Availability

Not applicable.

## Supplementary Materials

Supplementary materials are available online at https://www.mdpi.com/article/10.3390/ijms23031857/s1.
