# Supplementary material for: Metabolic Reprogramming in Response to Alterations of Mitochondrial DNA and Mitochondrial Dysfunction in Gastric Adenocarcinoma
Source: Int J Mol Sci. 2022 Feb 6;23(3):1857. doi: 10.3390/ijms23031857 (PMC8836428; doi:10.3390/ijms23031857)
Supplement: Supplementary file 1 [file ijms-23-01857-s001.zip › ijms-1471982-supplementary.pdf]

International Journal of *Molecular Sciences*

MDPI

Article

Supplementary Materials: Metabolic reprogramming in response to alterations of mitochondrial DNA and dysfunction of mitochondrial respiration in gastric adenocarcinoma

Tzu-Ching Chang, Hui-Ting Lee, Siao-Cian Pan, Shih-Han Cho, Chien Cheng, Liang-Hung Ou, Chia-I Lin, Chen-Sung Lin and Yau-Huei Wei

Table S1. Summary of the D310 alterations between the paired non-cancerous gastric mucosa (NC-GM) and the gastric adenocarcinoma (GAC) of 57 GAC patients

			D310 characteristics							
			Non-cancerous gastric mucosa (NC-GM)				Gastric adenocarcinoma (GAC)			
D310mutation	Types of D310 alterations	Patients	D310* variants	Variant number	Major variant	Pattern	D310* variants	Variant number	Major variant	Pattern
No (n=30)	Type I, Homoplasmic to homoplasmic (n=17)	GAC 01	7	1	7	Homoplasmy	7	1	7	Homoplasmy
		GAC 03	7	1	7	Homoplasmy	7	1	7	Homoplasmy
		GAC 04	7	1	7	Homoplasmy	7	1	7	Homoplasmy
		GAC 07	7	1	7	Homoplasmy	7	1	7	Homoplasmy
		GAC 09	7	1	7	Homoplasmy	7	1	7	Homoplasmy
		GAC 21	7	1	7	Homoplasmy	7	1	7	Homoplasmy
		GAC 25	7	1	7	Homoplasmy	7	1	7	Homoplasmy
		GAC 27	7	1	7	Homoplasmy	7	1	7	Homoplasmy
		GAC 29	7	1	7	Homoplasmy	7	1	7	Homoplasmy
		GAC 36	7	1	7	Homoplasmy	7	1	7	Homoplasmy
		GAC 41	7	1	7	Homoplasmy	7	1	7	Homoplasmy
		GAC 46	7	1	7	Homoplasmy	7	1	7	Homoplasmy
		GAC 48	7	1	7	Homoplasmy	7	1	7	Homoplasmy
		GAC 57	7	1	7	Homoplasmy	7	1	7	Homoplasmy
		GAC 60	7	1	7	Homoplasmy	7	1	7	Homoplasmy
		GAC 61	7	1	7	Homoplasmy	7	1	7	Homoplasmy
		GAC 62	7	1	7	Homoplasmy	7	1	7	Homoplasmy
	Type II, Heteroplasmic to heteroplasmic (n=13)	GAC 02	9,8	2	9	Heteroplasmy	9,8	2	9	Heteroplasmy
		GAC 08	8,7	2	8	Heteroplasmy	8,7	2	8	Heteroplasmy
		GAC 12	8,7	2	8	Heteroplasmy	8,7	2	8	Heteroplasmy
		GAC 14	8,7	2	8	Heteroplasmy	8,7	2	8	Heteroplasmy
		GAC 15	8,7	2	8	Heteroplasmy	8,7	2	8	Heteroplasmy
		GAC 18	8,9	2	8	Heteroplasmy	8,9	2	8	Heteroplasmy
		GAC 20	8,7	2	8	Heteroplasmy	8,7	2	8	Heteroplasmy
		GAC 28	8,9	2	8	Heteroplasmy	8,9	2	8	Heteroplasmy

		GAC 31	8,7	2	8	Heteroplasmy	8,7	2	8	Heteroplasmy
		GAC 34	8,9	2	8	Heteroplasmy	8,9	2	8	Heteroplasmy
		GAC 39	8,7,9	3	8	Heteroplasmy	8,7,9	3	8	Heteroplasmy
		GAC 53	8,7	2	8	Heteroplasmy	8,7	2	8	Heteroplasmy
		GAC 55	8,7	2	8	Heteroplasmy	8,7	2	8	Heteroplasmy
Yes (n=27)	Type III, Heteroplasmic to heteroplasmic (n=22)	GAC 05	8,9,7	3	8	Heteroplasmy	9,8,10	3	9	Heteroplasmy
		GAC 06	8,7	2	8	Heteroplasmy	8,7	2	8	Heteroplasmy
		GAC 10	9,8,10,7	4	9	Heteroplasmy	7,9	2	7	Heteroplasmy
		GAC 11	8,7	2	8	Heteroplasmy	8,7	2	8	Heteroplasmy
		GAC 13	8,7	2	8	Heteroplasmy	8,7,9	3	8	Heteroplasmy
		GAC 16	8,7	2	8	Heteroplasmy	8,7,9	3	8	Heteroplasmy
		GAC 17	8,7	2	8	Heteroplasmy	8,9,7	3	8	Heteroplasmy
		GAC 19	8,9,7	3	8	Heteroplasmy	7,8,9	3	7	Heteroplasmy
		GAC 22	8,7	2	8	Heteroplasmy	7,8	2	7	Heteroplasmy
		GAC 23	8,7,9	3	8	Heteroplasmy	8,10,9,7	4	8	Heteroplasmy
		GAC 24	9,8	2	9	Heteroplasmy	9,8,10	3	9	Heteroplasmy
		GAC 30	8,7	2	8	Heteroplasmy	8,7	2	8	Heteroplasmy
		GAC 32	8,7	2	8	Heteroplasmy	8,7	2	8	Heteroplasmy
		GAC 35	9,8	2	9	Heteroplasmy	9,8,10	3	9	Heteroplasmy
		GAC 38	7,8	2	7	Heteroplasmy	8,7	2	8	Heteroplasmy
		GAC 42	9,8	2	9	Heteroplasmy	8,9	2	8	Heteroplasmy
		GAC 43	8,7	2	8	Heteroplasmy	8,7,9	3	8	Heteroplasmy
		GAC 47	9,8,10	3	9	Heteroplasmy	9,8,10	3	9	Heteroplasmy
		GAC 52	8,7	2	8	Heteroplasmy	8,9	2	8	Heteroplasmy
		GAC 59	9,8,7,6	4	9	Heteroplasmy	9,8,10	3	9	Heteroplasmy
		GAC 63	9,8	2	9	Heteroplasmy	9,8	2	9	Heteroplasmy
		GAC 65	8,7	2	8	Heteroplasmy	8,9,6,7	4	8	Heteroplasmy
	Type IV, Heteroplasmic to homoplasmic (n=5)	GAC 26	8,7	2	8	Heteroplasmy	8	1	8	Homoplasmy
		GAC 51	8,7	2	8	Heteroplasmy	8	1	8	Homoplasmy
		GAC 54	7,8	2	7	Heteroplasmy	7	1	7	Homoplasmy
		GAC 56	8,7	2	8	Heteroplasmy	7	1	7	Homoplasmy
		GAC 64	7,6	2	7	Heteroplasmy	7	1	7	Homoplasmy

*The cytosine (C) numbers of D310 variants are listed in order according to the detected sequencing peaks of thymidine (T)

Non-cancerous gastric mucosa **Gastric adenocarcinoma**

Type I. Homoplamic to homoplasmic alteration – without D310 mutation (n=17)

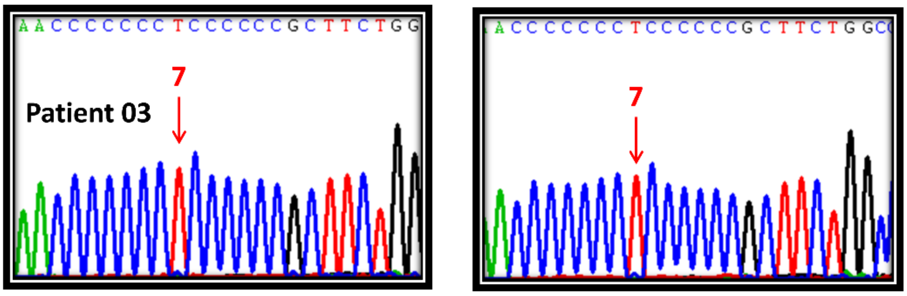

Type II. Heteroplasmic to heteroplasmic alteration – without D310 mutation (n=13)

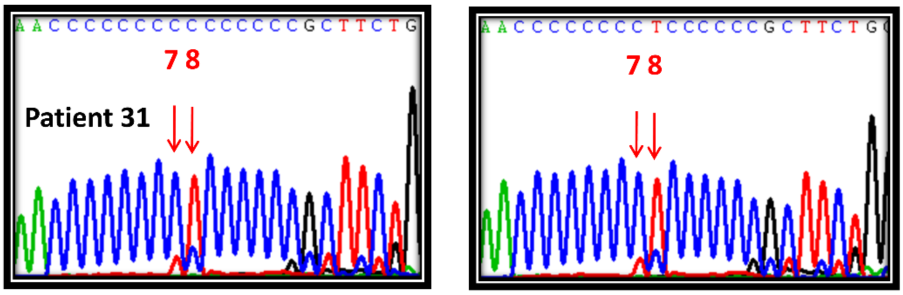

Type III. Heteroplasmic to heteroplasmic alteration – with D310 mutation (n=22)

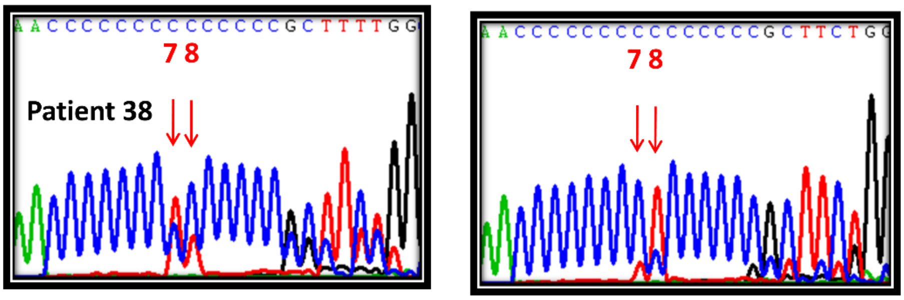

Type IV. Heteroplasmic to homoplasmic alteration – with D310 mutation (n=5)

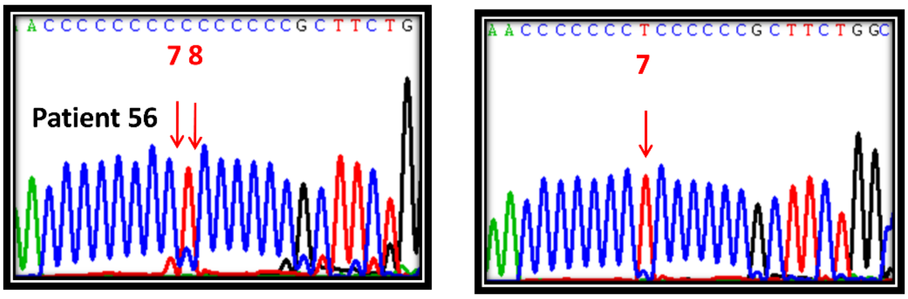

Figure S1. Types and representative cases to demonstrate the mitochondrial DNA D310 variants and their changes between NC-GM (left) and paired GAC (right). T (thymidine) is shown in red, A (adenine) in green, C (cytosine) in blue and G (guanine) in black during sequencing. The Arabic number above the red arrow denotes the C number before the indicated T peak and the T peak height represents the relative quantity of the D310 variant. Abbreviations: NC-GM, non-cancerous gastric mucosa; GAC, gastric adenocarcinoma.

Type I: Homoplasmic to homoplasmic alteration - without D310 mutation (n=17): Take patient 03 as an example. His NC-GM harbors one kind of D310 variant, the C_7TC_6, and is named as homoplasmic D310 with C_7TC_6 as the major one. His GAC also harbors one kind of D310 variant, the C_7TC_6, and is named as homoplasmic D310 with C_7TC_6 as the major one. No significant shift of the D310 sequence variations is noted. As a result, patient 03 is defined as homoplasmic to homoplasmic alteration without D310 mutation.

Type II: Heteroplasmic to heteroplasmic alteration - without D310 mutation (n=13): Take patient 31 as an example. His NC-GM harbors 2 kinds of D310 variants, the C_8TC_6 and C_7TC_6 in order, and is named as heteroplasmic D310 with C_8TC_6 as the major one. His GAC also harbors 2 kinds of D310 variants, the C_8TC_6 and C_7TC_6 in order, and is named as heteroplasmic D310 with C_8TC_6 as the major one. No significant shift of the D310 sequence variations is noted. As a result, patient 31 was defined as heteroplasmic to heteroplasmic alteration without D310 mutation.

Type III, Heteroplasmic to heteroplasmic alteration - with D310 mutation (n=22): Take patient 38 as an example. His NC-GM harbors 2 kinds of D310 variants, the C_7TC_6 and C_8TC_6 in order, and is named as heteroplasmic D310 with C_7TC_6 as the major one. His GAC harbors 2 kind of D310 variants, the C_8TC_6 and C_7TC_6 in order, and is named as heteroplasmic D310 with C_8TC_6 as the major one. Significant shift of the D310 sequence variations is noted. As a result, patient 38 is defined as heteroplasmic to heteroplasmic alteration with D310 mutation.

Type IV, Heteroplasmic to homoplasmic alteration - with D310 mutation (n=5): Take patient 56 as an example. His NC-GM harbors 2 kinds of D310 variants, the C_8TC_6 and C_7TC_6 in order, and is named as heteroplasmic D310 with C_8TC_6 as the major one. His GAC harbors one kind of D310 variant, the C7TC6, and is named as homoplasmic D310 with C_7TC_6 as the major one. Significant shift of the D310 sequence variations is noted. As a result, patient 56 is defined as heteroplasmic to homoplasmic alteration with D310 mutation.

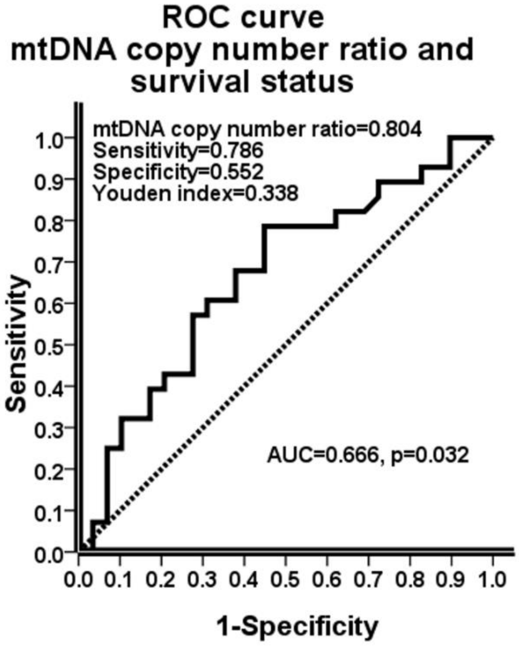

Figure S2. ROC curve (AUC=0.666, 95%CI=0.523-0.808, p=0.032) is plotted to figure out the optimal mtDNA copy number ratio to distinguish survival status of the 57 GAC patients. It demonstrates mtDNA copy number ratio of 0.804 harbors

the highest Youden index of 0.338 (Sensitivity=0.786, Specificity=0.552). ROC: receiver operating characteristic, AUC: area under the curve

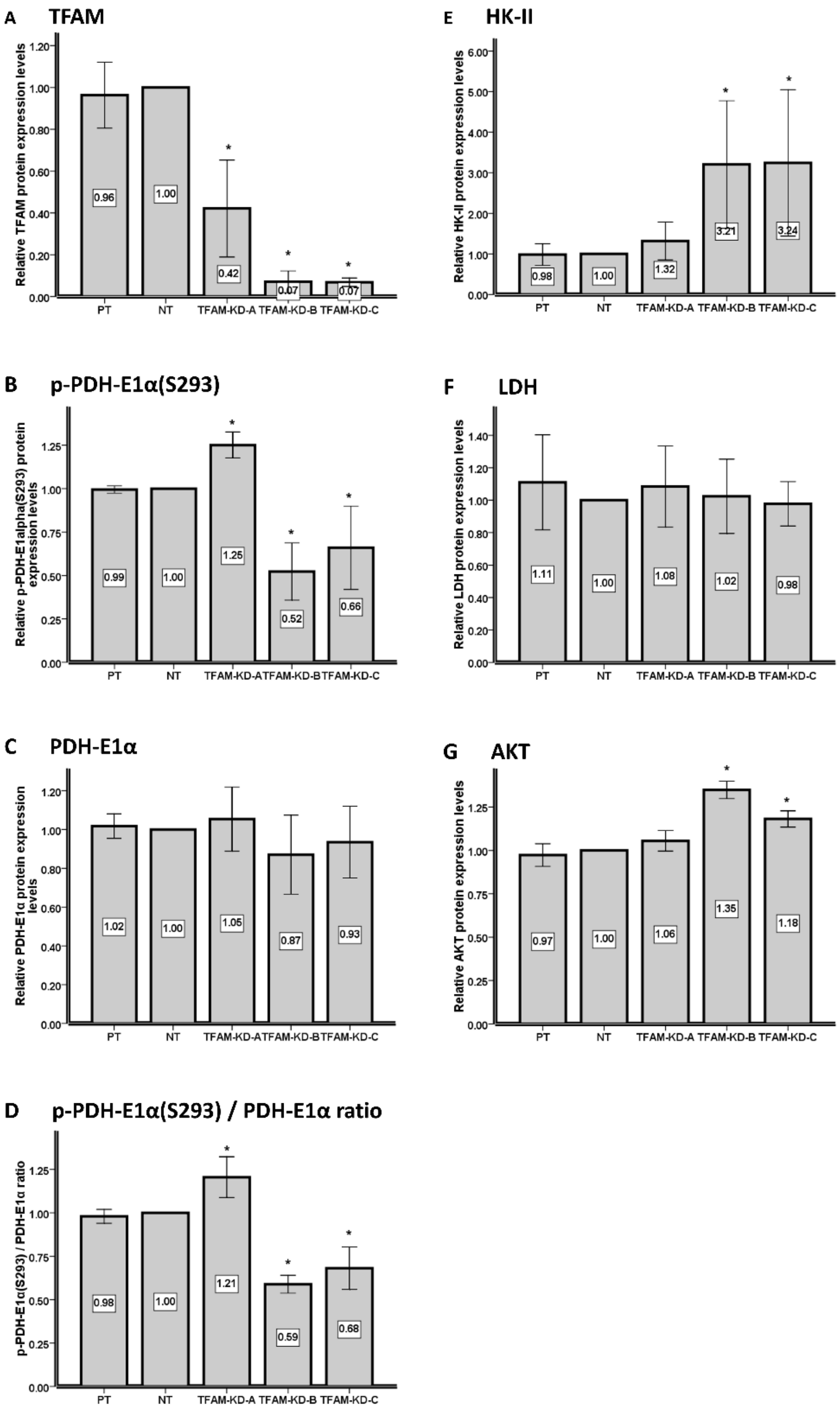

Figure S3. Relative protein expression levels of (A) TFAM, (B) p-PDH-E1α(S293), (C) PDH-E1α, (E) HK-II, (F) LDH and (G)AKT as well as (D) p-PDH-E1α(S293)/PDH-E1α ratios among PT, NT, TFAM-KD-A, TFAM-KD-B and TFAM-KD-C cells are shown. The expression level of NT is defined as 1.00. The symbol * denotes a significant difference is achieved as compared to NT (p<0.05). TAFM: mitochondrial transcription factor A; PDH-E1α: pyruvate dehydrogenase E1α sub-

unit; p-PDH-E1α (293): phosphorylated pyruvate dehydrogenase E1α subunit; HK-II: hexokinase II; LDH: lactate dehydrogenase; AKT: v-akt murine thymoma viral oncogene homolog 1 gene (*AKT*)-encoded AKT; NT: Null target; PT: Parental

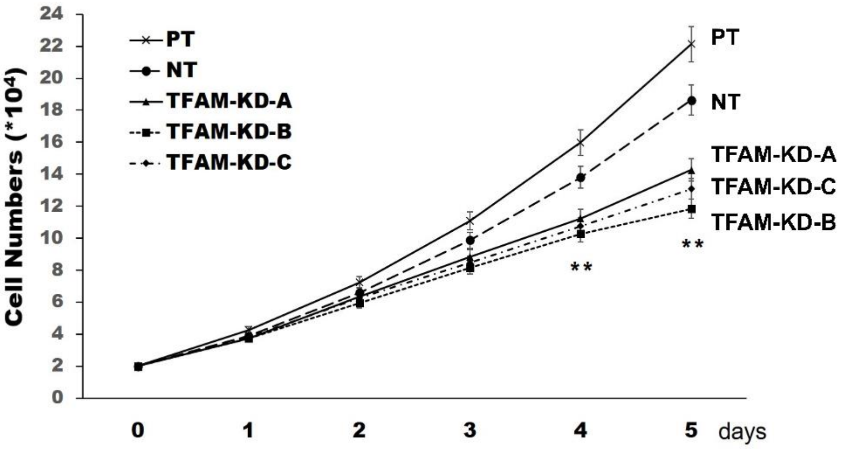

Figure S4. Cell proliferation rates of PT, NT, TFAM-KD-A, TFAM-KD-B and TFAM-KD-C cells are illustrated. An aliquot of 2 × 10^4 cells were plated in 35 mm culture dishes (day 0) and incubated in 5% CO2 at 37°C. Cells were incubated in culture medium with Puromycin (NT, TFAM-KD-A, TFAM-KD-B, and TFAM-KD-C cells) or without Puromycin (PT cells). Viable cells were determined with trypan blue (0.4%), and counted by Countess 3 Automated Cell Counter (Invitrogen) in triplicate on days 1, 2, 3, 4, and 5, and the cell proliferation curves were drawn (Mean±SD, N=3). The symbol ** denotes p<0.01 when the TFAM-KD-A/-B /-C are compared with NT, respectively. NT: Null target; PT: Parental

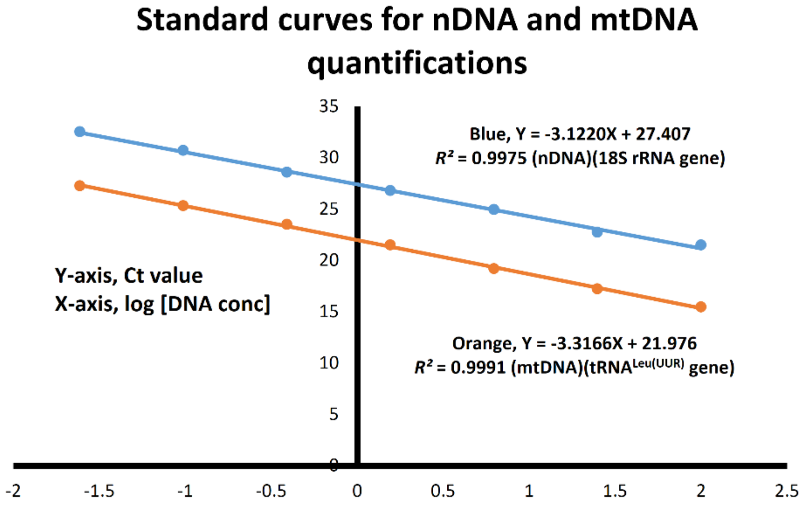

Figure S5. Standard curves for nDNA (R^2=0.9975) and mtDNA (R^2=0.9991) quantification are plotted. X and Y stand for log[DNA conc.] and Ct value, respectively Total cellular DNA of AGS cells were serially diluted 4-folded from 100 ng/μL (X=log100=2.00000) to 0.024414 ng/μL (X=log0.024414=-1.61236) for Q-PCR to detect Ct values of mtDNA and nDNA, respectively. Blue line represents for nDNA and orange line for mtDNA, respectively.
